# The Diversity and Distribution of Viruses Associated with *Culex annulirostris* Mosquitoes from the Kimberley Region of Western Australia

**DOI:** 10.3390/v12070717

**Published:** 2020-07-02

**Authors:** Simon H. Williams, Avram Levy, Rachel A. Yates, Nilusha Somaweera, Peter J. Neville, Jay Nicholson, Michael D. A. Lindsay, John S. Mackenzie, Komal Jain, Allison Imrie, David W. Smith, W. Ian Lipkin

**Affiliations:** 1 Center for Infection and Immunity, Mailman School of Public Health of Columbia University, New York, NY 10034, USA; ray2112@cumc.columbia.edu (R.A.Y.); kj2230@cumc.columbia.edu (K.J.); 2 School of Biomedical Sciences, Faculty of Health and Medical Sciences, University of Western Australia, Nedlands, WA 6009, Australia; allison.imrie@uwa.edu.au; 3 PathWest Laboratory Medicine WA, Nedlands, WA 6009, Australia; Avram.Levy@health.wa.gov.au (A.L.); j.mackenzie@curtin.edu.au (J.S.M.); 4 Environmental Health Directorate, Public and Aboriginal Health Division, Department of Health, Western Australia, Perth, WA 6010, Australia; Nilusha.Gunarathne@health.wa.gov.au (N.S.); peter.neville@health.wa.gov.au (P.J.N.); jay.nicholson@health.wa.gov.au (J.N.); michael.lindsay@health.wa.gov.au (M.D.A.L.); 5 Faculty of Health Sciences, Curtin University, Perth, WA 6012, Australia; 6 School of Chemistry and Molecular Biosciences, The University of Queensland, St Lucia, QL 4072, Australia

**Keywords:** *Culex annulirostris*, virome, Western Australia

## Abstract

Metagenomics revealed an impressive breadth of previously unrecognized viruses. Here, we report the virome of the *Culex annulirostris* Skuse mosquito, an important vector of pathogenic arboviruses in Australia. Mosquitoes were collected from three sites in the Kimberley region of Western Australia. Unbiased high-throughput sequencing (HTS) revealed the presence of 16 novel viral sequences that share less than 90% identity with known viruses. None were closely related to pathogenic arboviruses. Viruses were distributed unevenly across sites, indicating a heterogeneous *Cx. annulirostris* virome. Polymerase chain reaction assays confirmed HTS data and identified marked variation between the virus prevalence identified at each site.

## 1. Introduction

Mosquito-borne viral diseases pose a persistent challenge to global public health. Flaviviruses, such as yellow fever, dengue, Zika, Japanese encephalitis, and West Nile (WNV), and alphaviruses including Ross River and chikungunya cause significant morbidity and mortality in humans [1,2,3]. Each of these viruses was discovered through classical methods, including culture, electron microscopy, and serology [4]; however, recent metagenomic studies suggest they represent just a fraction of the complete mosquito virome [5,6,7,8].

Comprehensive arboviral surveillance was conducted in Western Australia (WA) since 1972 [9]. Field-caught mosquitoes are transported by cold chain from across WA to a centralized laboratory to be sorted [10], pooled, and processed for inoculation onto invertebrate and vertebrate cells [11]. Viral supernatant is collected from cultures displaying a cytopathic effect and characterized by enzyme immunoassay using a panel of monoclonal antibodies to Australian arboviruses [12]. Genomic characterization of an isolate can be carried out using PCR [9], high-throughput sequencing (HTS) [11], or a combination of both methods [13]. Since 2018, targeted screening using PCR mostly replaced virus culture and antigenic characterization for arboviral surveillance in WA. In addition, sentinel chicken serosurveillance is used for monitoring Murray Valley encephalitis virus (MVEV) and WNV (Kunjin) activity due to the difficulties in conducting regular mosquito collection in remote tropical and subtropical areas [14].

Here, we describe the virome of *Culex annulirostris* mosquitoes collected from three sites located in the Kimberley region in the far north of WA that are representative of locations where MVEV and WNV (Kunjin) are most regularly detected in surveillance programs and where human cases are most frequent [15]. Prior studies of viruses isolated from mosquitoes in WA detected novel arboviruses capable of infecting humans and animals [13,16]. Techniques such as HTS have the potential to detect known human or animal viruses, viruses closely related to them, or novel agents with pathogenic potential, without the need for laborious cell culture systems or the ethically challenging use of sentinel animals. This study was undertaken in order to evaluate the ability of HTS to identify the diversity of viruses present in mosquitoes, including the detection of current and possible future human and/or animal pathogens within a geographic area where important and emerging arboviruses threaten. *Cx. annulirostris* was chosen, as it is the major vector of the human pathogenic flaviviruses in this region, as well as a significant vector for pathogenic alphaviruses.

## 2. Materials and Methods

### 2.1. Mosquito Collection

**Figure 1:**
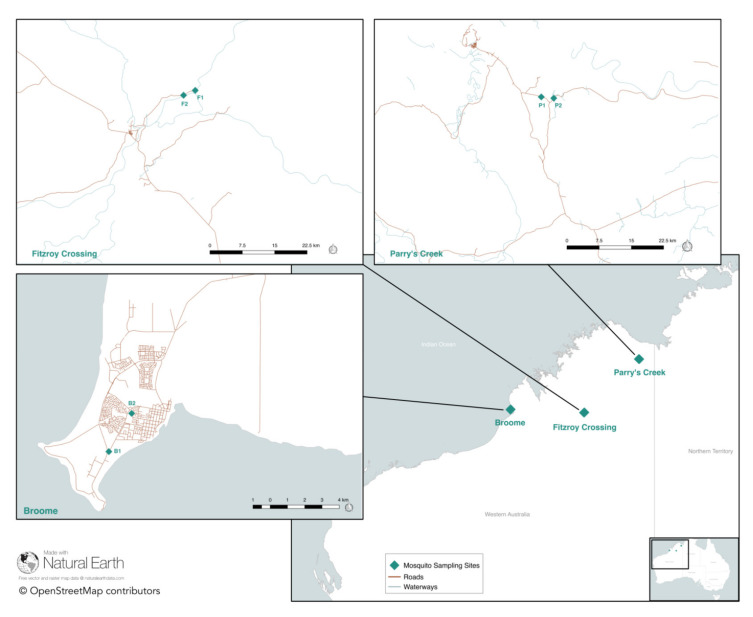
Map of northern Western Australia. Mosquito trap locations for three sites and six traps are marked with a teal diamond. The inset maps indicate the locations of two traps located at each site. The map was prepared using QGIS v2.18.15 [18], OpenStreetMap, Natural Earth, and Mainroads Western Australia [19]. The baselayer shapefile was obtained from the Australian Government data portal [20].

Adult mosquitoes were trapped using Encephalitis Virus Surveillance CO_2_-baited traps [17] as part of routine arboviral surveillance at three sites (Broome, Fitzroy Crossing, and Parry’s Creek) in the Kimberley region in the north of WA during March and April 2018 [15] (Figure 1, Table 1). Mosquitoes trapped from the townships of Broome and Fitzroy Crossing were collected from public land, while mosquitoes collected from the rotunda within Geikie Gorge National Park (Fitzroy Crossing) and from Parry Lagoons Nature Reserve (Parry’s Creek) were collected under Department of Parks and Wildlife, WA, permit number 08-001839-1. Two traps were located approximately 2.5 km apart at each site. Trapped mosquitoes were transported to laboratories located in Perth where they were sorted by species using a morphologic key [10]. A total of 20,556 *Cx. annulirostris* were trapped at the three sites used in this study (Table 1). Fifty *Cx. annulirostris* mosquitoes were set aside from each trap for processing, for a total of 300 mosquitoes. Blood-fed mosquitoes were excluded from further analysis.

### 2.2. Nucleic Acid Purification and High-Throughput Sequencing

Mosquitoes were individually washed three times using 750 µL of phosphate-buffered saline to remove environmental nucleic acids. Three 5-mm stainless-steel beads (Qiagen, Hilden, Germany) and 750 µL of cold virus transport medium (in-house formulation [21]) were added to each washed mosquito. Homogenization was performed using the TissueLyserLT (Qiagen, Hilden, Germany) set to 50 kHz for 5 min. For unbiased HTS, we pooled supernatants and enriched for virus particles. Aliquots of 50 µL of supernatant from each of the 25 homogenized individual mosquitoes were pooled according to trap for a total of 12 pools. An aliquot of 250 µL of pooled supernatants was passed through a 0.45-µM filter (EMD Millipore, Bedford, MA, USA), nuclease-treated (1.5 µL of RNase A (Invitrogen, Carlsbad, CA, USA), 1.8 µL of benzonase (EMD Millipore, Billerica, MA, USA), and 2.7 µL of 1 M MgCl_2_), and incubated at room temperature for 45 min. Total nucleic acid was extracted from pools using the MagMax Express-96 automated platform (Applied Biosystems, Foster City, CA, USA) with modifications [22]. Nucleic acid concentration and purity were measured on the NanoDrop 1000 spectrophotometer (Thermo Scientific, Wilmington, DE, USA). Total nucleic acid was reverse-transcribed using SuperScript III (Invitrogen) and treated with RNAse H (Invitrogen). Double-stranded complementary DNA (cDNA) was synthesized using Klenow fragment (3′–5′ exo-) (New England Biolabs, Beverly, MA, USA). Double-stranded cDNA was fragmented on the Focused-Ultrasonicator E210 (Covaris, Woburn, MA, USA). Libraries were uniquely barcoded and prepared for sequencing on a single lane of the HiSeq 4000 system (Illumina, San Diego, CA, USA) using the Hyper Prep kit (Kapa Biosystems, Boston, MA, USA). Two negative control libraries were prepared: the first to control nucleic acid extraction and library preparation (NTC-EL) and the second to control library preparation only (NTC-L). A positive control containing known agents (ERCC spike-in) was also included.

Sequencing reads generated from HTS processing were demultiplexed, trimmed, and quality-filtered using PRINSEQ v0.20.2 [23]. Sequences mapping to the only available *Culex* genome, *Cx. quinquefasciatus*, (National Center for Biotechnology Information (NCBI) reference sequence NZ_AAWU00000000.1) were subtracted using Bowtie2 (v2.0.6; [24]). Assembly of quality-filtered reads was performed using MEGAHIT (v1.0; [25]). Contiguous sequences and unique singletons were aligned against viral nucleotide or protein sequences contained within the non-redundant databases of Genbank using MegaBLAST (Basic Local Alignment Search Tool) and BLASTx. All single-end reads were mapped with Bowtie 2 (v2.0.6) against consensus genomic sequences for the 16 viruses identified using HTS. BAM files were parsed using BEDTools (v2.26.0), and perl scripts were used to obtain viral abundance. Virus-mapped reads were corrected for sample bleeding due to index cross-talk using a cutoff of 0.05% [26] and normalized relative to total reads for each pool. A heatmap was prepared in Microsoft Excel.

### 2.3. PCR Screening

Nucleic acids from the same 300 individual mosquitoes used in pooling experiments were purified from 250 µL of supernatant to determine the prevalence and distribution of six representative viruses identified from HTS analysis. Supernatants were processed using the same modified MagMax Express-96 platform (Applied Biosystems) protocol as described above. Complementary DNA was prepared from TNA using SuperScript III (Invitrogen) and random hexamer primers. PCR screening primers were designed using assembled viral sequences obtained from HTS data (Table 2). All PCR assays were performed using an annealing temperature of 60 °C followed by 10 cycles, decreasing by 0.5 °C per cycle, and a final annealing temperature of 55 °C was maintained for a further 35 cycles. All PCR products of the anticipated size were confirmed using Sanger sequencing.

### 2.4. Phylogenetics

RNA-dependent RNA polymerase (RdRp) sequences were utilized for phylogenetic analyses. Representative protein sequences were aligned using MUSCLE in Geneious 10.2.3 [27] and exported to MEGA6 [28] where best-fit model testing was performed. The Le and Gascuel substitution model [29] was employed for hepe-virga viruses, iflavirus, reo-like virus, toti- and chryso-like viruses, luteo-like virus, partiti-like virus, and densovirus analysis. The rtRev model [30] was used for phenuiviruses and qinvirus. Maximum likelihood phylogenetic analyses were prepared using 500 bootstrap repetitions. Newick trees were exported to Figtree (http://tree.bio.ed.ac.uk/software/figtree) for annotation.

### 2.5. Accession Numbers

Consensus viral genomic sequences were deposited in GenBank under accession numbers MT498812 to MT498834. Illumina sequence data were deposited in GenBank under BioProject identifier (ID) PRJNA632594. Illumina sequence data for Jogalong virus (accession number MN133813) were reported previously and are available under BioProject ID PRJNA590265.

## 3. Results

### 3.1. Mosquito Collection

*Culex annulirostris* was the most frequently captured mosquito species during the 2017–2018 season in the Kimberley region of WA [31]. Mosquitoes included in this study were described elsewhere [32].

### 3.2. High-Throughput Sequencing

A total of 341 million reads were obtained from a single lane of sequencing (excluding controls). An average of 10.5 million reads per mosquito pool, 2.4 million reads per negative control and 16.9 million for the positive control remained following primer trimming, quality-filtering, and host subtraction. Assembly of reads generated an average of 233,411 contigs (range: 133,941–430,368) and 2.7 million unique singletons per mosquito pool; 1.74% of these sequences shared identity with viral sequences using MegaBLAST and BLASTX similarity alignments with minimum E-value cutoffs of 1 × 10^−10^ and 1 × 10^−3^, respectively.

### 3.3. Virus Genomic Characterization

Analysis of 300 *Cx. annulirostris* revealed one single-stranded DNA (ssDNA), five positive-sense single-stranded RNA (ssRNA), four negative-sense ssRNA, and six double-stranded RNA (dsRNA) viral sequences (Table 3). Sequences represent six classified and five unclassified viral families. The majority of viral sequences identified in this study were RNA viruses (15/16) with a single DNA genome identified belonging to family *Parvoviridae*, sub-family *Densovirinae*. Six viruses shared less than 50% amino acid identity with their closest viral relative, indicating a breadth of viral diversity in *Cx. annulirostris* mosquitoes from this region. Only one virus (Jogalong virus, family *Flaviviridae*, genus *Hepacivirus*) shared identity with viruses typically associated with vertebrates. All other viruses shared phylogenetic relationships with invertebrate, fungal, or plant viruses.

#### 3.3.1. Positive-Sense ssRNA Viruses

##### Flavivirus

We obtained sequences distantly related to hepaciviruses from a single pool from the Parry’s Creek site. Phylogenetic analysis, PCR prevalence, genome annotation, and potential host association with *Podargus strigoides* (tawny frogmouth) for the sequence, tentatively named Jogalong virus, were previously described [32].

##### Nege- and Virgaviruses

**Figure 2:**
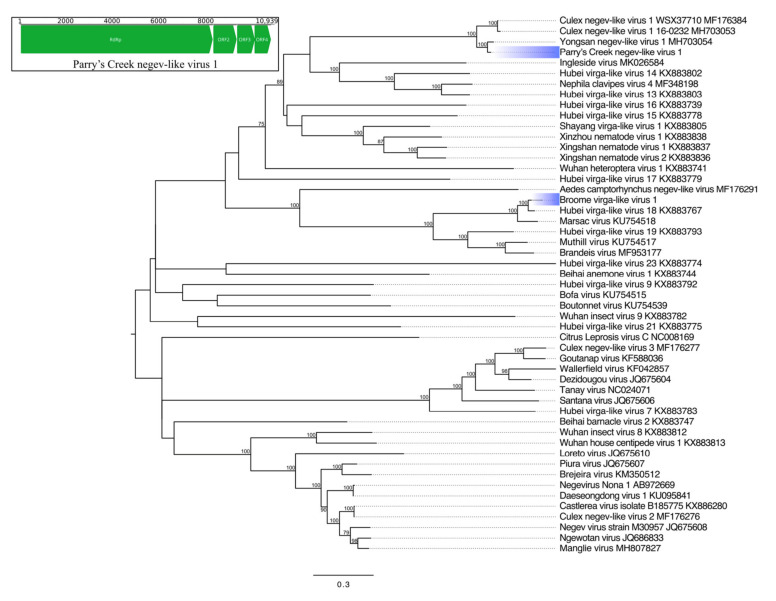
Phylogenetic analysis of Hepe-Virga viruses. The maximum likelihood tree was constructed using RdRp protein sequences. The scale bar represents units of substitutions per site, and bootstrap support values are displayed when greater than 70%. Viruses identified in this study are highlighted in blue. Inset: Genome organization of Parry’s Creek negev-like virus 1. Open reading frames (ORF) are indicated by green arrows, while nucleotide positions are shown above the genome. RdRp, RNA-dependent RNA polymerase.

Negeviruses have positive-sense ssRNA genomes and were first described in 2013 as widely distributed insect-specific viruses [34]. We identified a 10,939-nucleotide (nt) sequence, tentatively named Parry’s Creek negev-like virus 1 (PCNegV1) possessing an RdRp (2742 amino acids (aa)) with 88% aa identity to Yongsan negev-like virus 1 (YNegV1), a partially sequenced virus detected in *Cx. inatomii* from South Korea (Table 3). Phylogenetic analysis of the RdRp places PCNegV1 in a monophyletic clade shared with YNegV1 and two strains of Culex negev-like virus 1 (CxNegV1) detected in *Cx. australicus* (Australia [5]) and *Cx. pipiens* (Korea) (Figure 2). Aside from the RdRp, PCNegV1 possesses three further complete open reading frames (ORFs) at the 3′ end of the genome, whereas CxNegV1 possesses two ORFs, named hypothetical proteins 2 and 3; sequence outside of the YNegV1 RdRp was not obtained. When aligned, the position of the PCNegV1 ORF3 is nested between hypothetical proteins 2 and 3 of CxNegV1. The corresponding region in CxNegV1 contains a noncoding sequence. Interestingly, a single base pair substitution located at the 5′ end of this region (A → G, nt position 9300) for CxNegV1 (MH703053) restores a 252-aa ORF. This hypothetical peptide shares 52% identity with the corresponding protein encoded by the PCNegV1 ORF3. Together, these data could indicate a loss or gain of a peptide in these related viruses. PCNegV1 was detected by PCR from all three sites with an overall prevalence of 2.3% (Table 4).

Virgaviruses also possess positive-sense ssRNA genomes and belong to the same insect-infecting alphavirus-like superfamily as negeviruses [35]. An 11,189-nt contig encoding five complete ORFs was identified in one HTS pool from Broome (B1), tentatively named Broome virga-like virus 1. This sequence shares the greatest identity and a common genome architecture with a virus detected in flies in China, Hubei virga-like virus 18 (HVLV18) (33) (Table 3). All ORFs share greater than 80% aa identity with HVLV18: RdRp (82% aa identity), ORF2 (81%), ORF3 (89%), ORF4 (90%), and ORF5 (91%).

##### Iflavirus

Sequences related to picornaviruses were identified in a single pool from Fitzroy Crossing. The genome for Fitzroy Crossing iflavirus 1 (FCIflaV1) encodes a single 8718-nt open reading frame (2904 aa) and includes a 5′ nontranslated (NTR) region of 736 nt and a 139 nt 3′ NTR at each terminus. FCIflaV1 shares greatest similarity (48%) with Hubei arthropod virus 1, a member of family *Iflaviridae* that was sequenced from water striders, *Tetragnatha maxillosa* (spiders) and an arthropod mix in China [33] (Figure S1, Supplementary Materials; Table 3). PCR screening identified a single positive mosquito from Fitzroy Crossing (Table 4).

##### Luteo-Like Viruses

Luteoviruses were historically associated with plants [36], but were recently detected in arthropods (including mosquitoes [5]), molluscs, nematodes, and protists [33]. We obtained two partial segments from a virus tentatively named Broome luteo-like virus 1, which include two ORFs on RNA1 (RdRp and a hypothetical protein) and a single ORF on RNA2 (capsid). The ORFs on RNA1 share the highest identity with Culex mosquito virus 6 (RdRp, 77%; hypothetical protein, 46%; Figure S2, Supplementary Materials) and the remaining capsid protein in RNA2 is most similar to Culex mosquito virus 3 (70%) (Table 3). Culex mosquito viruses 6 and 3 were identified in California, United States of America (USA) and only single segments are available on GenBank for comparison [6].

#### 3.3.2. Negative-Sense ssRNA Viruses

##### Phenuiviruses

**Figure 3:**
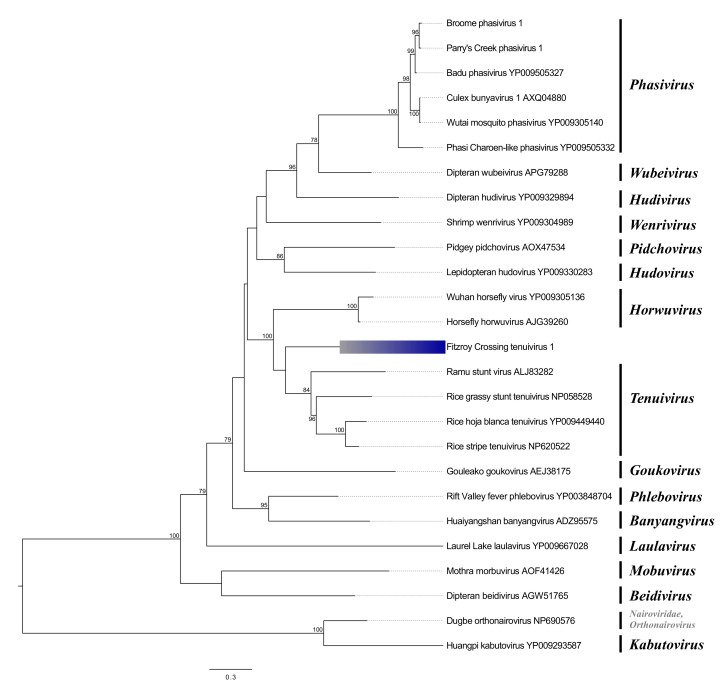
Phylogenetic analysis of phenuiviruses. The maximum likelihood tree was constructed using RdRp protein sequences. The scale bar represents units of substitutions per site and bootstrap support values are displayed when greater than 70%. Viruses identified in this study are highlighted in blue.

Three phenuiviruses representing two genera, *Phasivirus* and *Tenuivirus*, were recovered from across the three sites. Broome phasivrus 1 (BrPhasV1) and Parry’s Creek phasivirus 1 (PCPhasV1) share 87.5% and 88.3% aa identity in the RdRp protein, respectively, with Badu phasivirus (BADUV), an insect-specific bunyavirus identified in *Cx. annulirostris* and *Cx. sitiens* (Weidermann) mosquitoes [37] (Table 3). BADUV was originally isolated in Badu Island located in the Torres Strait approximately 2300 km to the northeast of Broome. BrPhasV1 and PCPhasV1 share 83% and 81% aa identity with the BADUV glycoprotein, as well as 80% and 82% with the BADUV nucleocapsid, respectively. Alignments between both *Cx. annulirostris* phasiviruses from WA reveal 91%, 88%, and 89% identity across the RdRp, glycoprotein, and capsid genes, respectively. The distribution of the phasiviruses across the three trapping sites was varied. BrPhasV1 was detected in 59% of mosquitoes in Broome, whereas it was rarely detected at the Fitzroy Crossing (2%) or Parry’s Creek (3%) sites (Table 4). Conversely, PCPhasV1 was not detected in Broome, while it was detected in a single mosquito in Fitzroy Crossing (1%); however, it was most prevalent in Parry’s Creek (16%) (Table 4). Phylogenetic analysis of the RdRp protein identified a common clade for both phasiviruses and with three other mosquito-associated phasiviruses: BADUV, Culex bunyavirus (*Culex* spp., USA [6]), and Wutai mosquito phasivirus (*Cx. quinquefasciatus* (Say), China [38]) (Figure 3).

The second phenuivirus, Fitzroy Crossing tenui-like virus 1 (FCTenV1), is highly divergent and shares just 33% aa identity with rice grassy stunt virus across the conserved RdRp protein (Table 3). Tenuiviruses typically possess between four and six segments, and they are plant-infecting viruses that are transmitted by vector planthoppers [39]. We were able to identify four coding regions representing the RdRp, glycoprotein (most similar to Wuchang cockroach virus 1; 31% aa identity), nonstructural protein NS4 (maize stripe tenuivirus; 25%), and nucleoprotein (Wuhan millipede virus 1; 31%). All ORFs share identity with plant tenuiviruses or bunyaviruses that were recently discovered as part of a comprehensive analysis of the arthropod virome [38]. PCR screening for FCTenLV1 indicated that the virus was present only in Fitzroy Crossing with a prevalence of 4% (Table 4). Phylogenetic analysis of the RdRp revealed that FCTenLV1 is rooted in a posterior position relative to the genus *Tenuivirus* (Figure 3).

##### Qinvirus

Qinviruses are a recently described and highly divergent clade of bisegmented negative-stranded ssRNA viruses associated with arthropods [33]. We identified a 5599-nt sequence, tentatively named Fitzroy Crossing qinvirus 1 (FCqinV1), encoding a 1752-aa ORF that shares greatest identity with Vinslov virus RdRp protein (80%), identified in *Cx. pipiens* from Sweden [40] (Table 3). Phylogenetic analysis of the RdRP protein supports sequence identity results; FCqinV1 shares a common node with Vinslov virus. Another qinvirus identified in *Cx. globocoxitis* (Dobrotworsky) from southern regions of WA, Wilkie qin-like virus, was only distantly related (32% aa identity in RdRp) to FCqinV1 (Figure S3, Supplementary Materials). However, there are limited published qinvirus sequences available for comparison, and the phylogenetic position of FCqinV1 is, thus, likely to change.

#### 3.3.3. Double-Stranded RNA Viruses

Six double-stranded RNA viruses were identified from across all trapping sites. Sequences share sequence similarity with reoviruses (Broome reo-like virus 1), totiviruses (Fitzroy Crossing toti-like viruses 1 and 2, Parry’s Creek toti-like virus 1), chrysoviruses (Broome chryso-like virus 1), and partitiviruses (Broome partiti-like virus 1). Broome reo-like virus 1 forms a sister clade to fijiviruses, but shares just 37% identity with Sanxia reo-like virus 1—an unclassified sequence identified in water strider from China [33] (Figure S4, Supplementary Materials; Table 3). The Fitzroy Crossing toti-like virus 1 RdRp protein sequence shared greatest identity (79%) with Lindangsbacken virus that was recently detected in *Cx. torrentium* in Sweden [40] (Figure S5, Supplementary Materials; Table 3). Parry’s Creek toti-like virus 1 also shared greatest identity with a virus detected in *Culex* mosquitoes from Sweden (Ahus virus, 75% identity) [40]. Fitzroy Crossing toti-like virus 2 and Broome chryso-like virus 1 were most similar to recently described arthropod-associated viruses from China [33] (Figure S5, Supplementary Materials; Table 3). Broome partiti-like virus 1, the only partiti-like virus recovered in this study shared greatest identity with a virus recovered from the Asian hornet (Vespa velutina partiti-like virus 1), but shares a clade with Hubei odonate virus 13 (Figure S6, Supplementary Materials; Table 3).

#### 3.3.4. Single-Stranded DNA Virus

**Figure 4:**
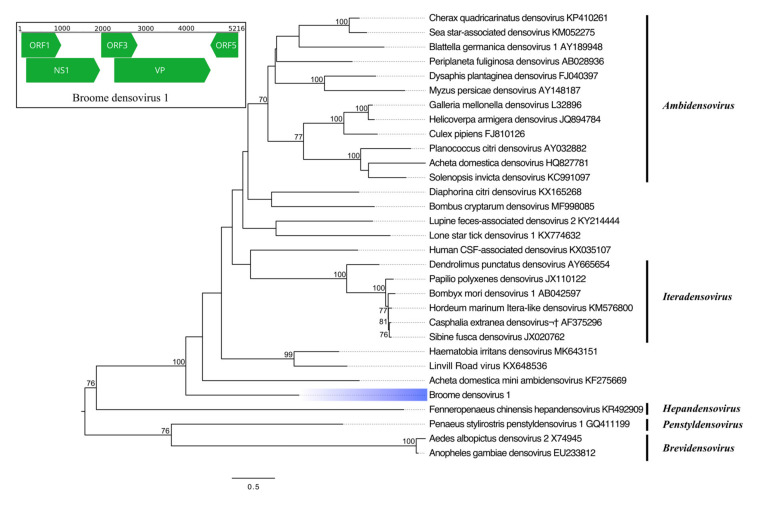
Phylogenetic analysis of Broome densovirus 1. The maximum likelihood tree was constructed using NS1 protein sequences. The scale bar represents units of substitutions per site, and bootstrap support values are displayed when greater than 70%. Viruses identified in this study are highlighted in blue. Inset: Genome organization of Broome densovirus 1. Open reading frames (ORF) are indicated by green arrows, while nucleotide positions are shown above the genome. NS1, non-structural protein 1; VP, viral capsid protein.

We identified a highly divergent densovirus, tentatively named Broome densovirus 1 (BrDenV1) from mosquitoes found at all three sites. BrDenV1 shares just 28% aa identity with Haematobia irritans densovirus (Table 3). Phylogenetic analysis of the NS1 protein places BrDenV1 within the diversity of densoviruses, but on a monophyletic clade deeply rooted in a posterior position to the *Iteradensovirus* and *Ambidensovirus* genera, as well as several unclassified viruses (Figure 4). The BrDenV1 genome consists of two large ORFs (NS1, 583 aa; VP, 760 aa), as well as two shorter ORFs (ORF1, 312 aa; ORF3, 285aa; ORF5, 218aa). ORF5 is in an ambisense orientation relative to all other ORFs.

### 3.4. Virus Distribution and Prevalence

**Figure 5:**
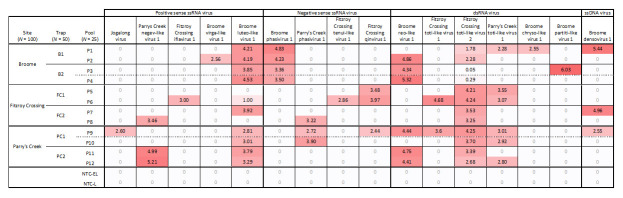
Heat map of HTS reads mapped against viral sequences found in *Cx. annulirostris* mosquitoes. Values represent the log_10_ of specific virus-mapped reads per 10,000,000 reads normalized against total reads per pool.

Raw sequencing reads were mapped to the 16 viral sequences discovered from HTS analysis of 300 *Cx. annulirostris* mosquitoes to assess the distribution across the three collection sites (Figure 5). We found a minimum of nine viral sequences at each site. Four viruses were detected at all sites (Broome luteo-like virus 1, Fitzroy Crossing toti-like virus 2, Parry’s Creek toti-like virus 1, and Broome densovirus 1), whereas seven viral sequences were each identified at one site. No viral sequences were identified in negative control libraries following normalization and correction for bleeding due to Illumina index cross talk.

To assess read abundance as a measure for viral distribution and prevalence, we performed PCR screening for six viruses (three positive-sense ssRNA and three negative-sense ssRNA) using individual mosquitoes. We confirmed the presence of each viral sequence in individual mosquitoes sourced from sites where HTS data indicated the presence of a virus sequence. We detected viral sequences by PCR in mosquitoes from pools on six occasions where HTS did not indicate the presence of the virus. This is likely due to several factors, including a dilution effect when pooling mosquitoes for HTS analysis and superior sensitivity of PCR versus unbiased HTS.

Three viruses screened by PCR were sparsely distributed with low abundance. Jogalong virus (0.3%), Fitzroy Crossing iflavirus 1 (0.3%), and Fitzroy Creek tenui-like virus 1 (0.7%) were detected at single sites in two or fewer mosquitoes (Table 4). The two phasiviruses, BrPhasV1 and PCPhasV1, were the most prevalent of all screened viruses and were identified in 21.3% and 5.7% of mosquitoes, respectively (Table 4).

## 4. Discussion

Arbovirus discovery and surveillance saw three major generational shifts over the last six decades. The use of suckling mice in the 1970s and 1980s led to the first wave of arbovirus discovery, including the isolation of Kununurra and Kimberley viruses from the northwest of WA [41]. A shift away from suckling mice to cell culture methods saw a reduction in the number and diversity of novel viruses, likely due to more selective conditions. However, the recent implementation of HTS approaches saw the catalog of mosquito-associated viruses expand once again.

The *Cx. annulirostris* mosquito is associated with the transmission of several important Australian arboviruses, including MVEV, Ross River virus, and WNV (Kunjin). To further investigate the complete virome of *Cx. annulirostris*, we performed HTS on 300 mosquitoes collected from across three sites in northern Western Australia. A diverse collection of 16 novel viral sequences was recovered, representing 11 distinct classified and unclassified viral families. We did not recover any sequences with homology to known human or mammalian pathogens. One sequence, Jogalong virus, shares identity with vertebrate-associated hepaciviruses, and we presented evidence elsewhere that this incidental discovery may be due to contamination from a blood meal taken from the tawny frogmouth (*Podargus strigoides*) [32]. However, we did demonstrate the capacity to detect a diverse array of RNA and DNA viruses including highly divergent viruses related to bunyaviruses, flaviviruses, picornaviruses, reoviruses, and parvoviruses. Therefore, although no sequences from known pathogenic arboviruses were recovered, we are confident that they would have been recovered if they were present.

Previous studies also showed that pathogenic viruses are rarely detected by HTS of mosquito samples [8,40,42]; thus, the lack of clinically important arboviruses discovered in our study was not unexpected. However, it was important to confirm this within the northern WA ecosystem. A metatranscriptomic investigation of 519 mosquitoes comprising five species, including three *Culex* species, from the southwest of WA recovered an impressive array of RNA viruses, but none that share identity with known pathogens [5]. A metagenomic analysis of over 12,000 *Culex* mosquitoes from California, USA, also failed to identify any known animal or human arboviruses despite the recovery of 56 mosquito-associated viral genomes [6]. Thus, it is possible that carriage of vertebrate pathogens by mosquitoes is rare or intermittent, and these viruses represent just a fraction of the mosquito virome. We were able to successfully demonstrate the diversity of the *Cx. annulirostris* virome, but our study and the unbiased metagenomic studies described above highlight the limitations of HTS for the purposes of surveillance for human and animal pathogenic arboviruses. Increased mosquito pool sizes and incorporation of positive selection systems to offset the impact of significant dilution effects may yield improved data for that purpose. Conversely, it does suggest that the detection of any pathogenic arboviruses in a mosquito population indicates an increased risk to human and animal health.

We identified differences between the viromes obtained from *Cx. annulirostris* at each collection site. Nine to 10 viruses were detected at each site, but the composition of the virome demonstrated variation. Aside from four viruses that were present across all three sites, seven viral sequences were confined to a single site and the remaining five viruses were confined to two sites. Phasivirus sequences that were 80% similar to Badu phasivirus and 90% similar to each other were identified from all sites. However, Broome phasivirus 1 was highly prevalent (59%) in mosquitoes sampled from Broome, whereas Parry’s Creek phasivirus was most prevalent (16%) in Parry’s Creek. Interestingly, each virus was either absent or rarely detected at the other site, while both phasiviruses were present in Fitzroy Crossing (located between Broome and Parry’s Creek), albeit in low prevalence (less than 4% for both viruses). These data indicate that even closely related viruses are distributed unevenly in *Cx. annulirostris* from across the north of WA. Differences between the local environments from each site may contribute to this unevenness [40]. Trapping locations differ climatically, by their proximity to water bodies and human settlements, and by their surrounding vegetation. Each of these factors may impact mosquito-breeding behaviors and indirectly contribute to differences observed in viral ecology. The differences observed between the *Cx. annulirostris* populations at each site suggest that there is minimal movement of mosquitoes across the remote Kimberley region of WA, as we would expect increased virus sharing and homogeneity if this were the case. However, longitudinal studies will be required to further investigate this finding.

We did not identify any viral sequences that share greater than 90% amino-acid identity across conserved RdRp proteins to known viruses. The mosquito taxon was proposed as playing an important role in virome structure [5], but we did not find evidence to support this at a genus level. Viruses obtained from three *Culex* species from the south of WA [5] shared little resemblance to the *Cx. annulirostris*-associated viruses identified in this study. Further virome analyses of other mosquito species from the same regions investigated in this study will be required to confirm this finding.

Arbovirus surveillance historically relied on pooling mosquitoes in order to determine the viral content at a community level. The proportion of viruses recovered from these mosquito pools is known as the infection rate [43]. Pooling is performed because of the impracticality of processing individual mosquitoes, especially when using classical virus propagation methods for large numbers of mosquitoes. Thus, the true prevalence of mosquito-associated viruses is rarely calculated on an individual mosquito level. Here, we performed PCR on individually processed mosquitoes to assess virus prevalence and compare to data obtained from the same collection of mosquitoes that were pooled for HTS. Overall, we found strong concordance between the presence of viral sequences from HTS pools and the detection of the same virus by PCR. However, virus PCR prevalence varied markedly and was not indicated by the number of HTS reads that mapped to a given virus genome. Thus, HTS performed using pooled samples provides an accurate qualitative assessment for the presence of a virus within mosquito populations represented in that pool; however, it is only a proxy for the true prevalence. This needs to be taken into consideration where HTS is used to detect potentially low-prevalence viruses, including known human pathogens. To maximize detection of those viruses, primary or supplemental testing by targeted PCRs should be used.

## Figures and Tables

**Table 1:** *Culex annulirostris* trapped from three sites in the Kimberley region. GPS—global positioning system.

Trap Code	Trap Location	GPS (Latitude)	GPS (Longitude)	Trap Set Date	Total Trapped *Cx. annulirostris*
B1	Adjacent to caravan park, Broome	−17.97763161	122.2125935	9 April 2018	188 *
B2	Cemetery, Broome	−17.95775700	122.2244490	10 April 2018	405 *
FC1	Rotunda, Fitzroy Crossing	−18.10542047	125.7016503	6 April 2018	135
FC2	Floodway, Fitzroy Crossing	−18.11554849	125.6773817	6 April 2018	170
PC1	Jogalong Billabong, Parry’s Creek	−15.59154698	128.2619530	26 March 2018	1971 *
PC2	Mangrove, Parry’s Creek	−15.59409900	128.2880270	26 March 2018	17,687 *

* Total *Cx. annulirostris* extrapolated based on species identification performed on 600 total mosquitoes per trap.

**Table 2:** PCR primers.

Agent	Target Gene	Forward Primer (5′–3′)	Reverse Primer (5′–3′)	Product Size (nt)
Jogalong virus	NS5B	CAGGTCCCTATTCTTACACGG	TCTGGTAACCGAGGTGTTGC	337
Broome phasivirus 1	RdRp	TTCAGATGGATTAAACCTGGCG	CTAGATCTTCTTGCCACTTCAGC	269
Parry’s Creek phasivirus 1	RdRp	CCAGACTGTTAGCAGCATCAATC	TCAATTCCTCTTGCCTGGAGAG	225
Fitzroy Crossing tenuivirus 1	RdRp	CTGGCATTGCCGGATTATCC	CTAGGCTTGAAATGACTCCAGG	351
Parry’s Creek negev-like virus 1	RdRp	AACTGCAGAGGGTGATATCGG	ATAGCATCGCCGCTCTTCC	204
Fitzroy Crossing iflavirus 1	Polyprotein	GTTGCAATACTACCAACGGCTC	CAAACCCACCATCGTGGTC	240

Nt, nucleotide; RdRp, RNA-dependent RNA polymerase; NS5B, non-structural protein 5B.

**Table 3:** Viruses detected from pools of virus-particle purified *Culex annulirostris* by unbiased high-throughput sequencing (HTS). BLAST—Basic Local Alignment Search Tool.

Virus Name	Family	BLAST Region	BLAST Length (aa)	Most Similar Viral Sequence	Coverage (%)	BLAST Identity (%)	E-Value
Jogalong virus	*Flaviviridae*	Polyprotein	2941	Bald eagle hepacivirus	91	40.32	0
Parry’s Creek negev-like virus 1	Hepe-Virga *	RdRp	2742 *	Yongsan negev-like virus 1	99 *	87.71	0
Broome virga-like virus 1	Hepe-Virga *	RdRp	2417	Hubei virga-like virus 18	100	81.81	0
Fitzroy Crossing iflavirus 1	*Iflaviridae*	Polyprotein	2905	Hubei arthropod virus 1	96	47.74	0
Broome luteo-like virus 1	Luteo-Sobemo *	RdRp	372	Culex mosquito virus 6	100	76.88	0
Broome phasivirus 1	*Phenuiviridae*	RdRp	2182	Badu phasivirus	100	87.53	0
Parry’s Creek phasivirus 1	*Phenuiviridae*	RdRp	2219	Badu phasivirus	99	88.27	0
Fitzroy Crossing tenui-like virus 1	*Phenuiviridae*	RdRp	2842	Rice grassy stunt virus	78	32.98	0
Fitzroy Crossing qinvirus 1	Qinvirus *	RdRp	1752	Vinslov virus	100	79.74	0
Broome reo-like virus 1	*Reoviridae*	RdRp	1308	Sanxia reo-like virus 1	88	36.76	9 × 10^−177^
Fitzroy Crossing toti-like virus 1	Toti-Chryso *	RdRp	806	Lindangsbacken virus	100	79.16	0
Fitzroy Crossing toti-like virus 2	Toti-Chryso *	Hyp. protein 2	758	Hubei toti-like virus 10	89	49.93	0
Parry’s Creek toti-like virus 1	Toti-Chryso *	RdRp	1418	Ahus virus	91	75.48	0
Broome chryso-like virus 1	*Chrysoviridae*	RdRp	1118	Hubei chryso-like virus 1	100	88.46	0
Broome partiti-like virus 1	Partiti-Picobirna *	RdRp	393	Vespa velutina partiti-like virus 1	96	56.81	1 × 10^−149^
Broome densovirus 1	*Parvoviridae*	NS1	583	Haematobia irritans densovirus	86	28.04	2 × 10^−53^

* Proposed clade [33]; RdRp, RNA-dependent RNA polymerase; aa, amino acid.

**Table 4:** PCR screening of individual *Culex annulirostris* mosquitoes for viruses identified by HTS of pooled samples.

Site	Trap	Jogalong Virus	Parry’s Creek Negev-Like Virus 1	Fitzroy Crossing Iflavirus 1	Broome Phasivirus 1	Parry’s Creek Phasivirus 1	Fitzroy Crossing Tenui-Like Virus 1
Broome	B1	0/50	0/50	0/50	35/50 (70%)	0/50	0/50
	B2	0/50	1/50 (2%)	0/50	24/50 (48%)	0/50	0/50
Fitzroy Crossing	FC1	0/50	0/50	1/50 (2%)	2/50 (4%)	0/50	2/50 (4%)
	FC2	0/50	1/50 (2%)	0/50	0/50	1/50 (2%)	0/50
Parry’s Creek	PC1	1/50 (2%)	2/50 (4%)	0/50	2/50 (4%)	14/50 (28%)	0/50
	PC2	0/50	3/50 (6%)	0/50	1/50 (2%)	2/50 (4%)	0/50
Total		1/300 (0.3%)	7/300 (2.3%)	1/300 (0.3%)	64/300 (21.3%)	17/300 (5.7%)	2/300 (0.7%)

## Supplementary Materials

The following are available online at https://www.mdpi.com/1999-4915/12/7/717/s1: Figure S1. Phylogenetic analysis of Fitzroy Crossing iflavirus 1; Figure S2. Phylogenetic analysis of Broome luteo-like virus 1; Figure S3. Phylogenetic analysis of Fitzroy Crossing qinvirus 1; Figure S4. Phylogenetic analysis of Broome reo-like virus 1; Figure S5. Phylogenetic analysis of chryso-like and toti-like viruses; Figure S6. Phylogenetic analysis of Broome partiti-like virus 1.
