# Supplementary material for: The Diversity and Distribution of Viruses Associated with Culex annulirostris Mosquitoes from the Kimberley Region of Western Australia
Source: Viruses. 2020 Jul 2;12(7):717. doi: 10.3390/v12070717 (PMC7411826; doi:10.3390/v12070717)
Supplement: Supplementary file 1 [file viruses-12-00717-s001.pdf]

viruses

Article

The Diversity and Distribution of Viruses Associated with *Culex annulirostris* Mosquitoes from the Kimberley Region of Western Australia

Simon H. Williams, Avram Levy, Rachel A. Yates, Nilusha Somaweera, Peter J. Neville, Jay Nicholson, Michael D.A. Lindsay, John S. Mackenzie, Komal Jain, Allison Imrie, David W. Smith, W. Ian Lipkin

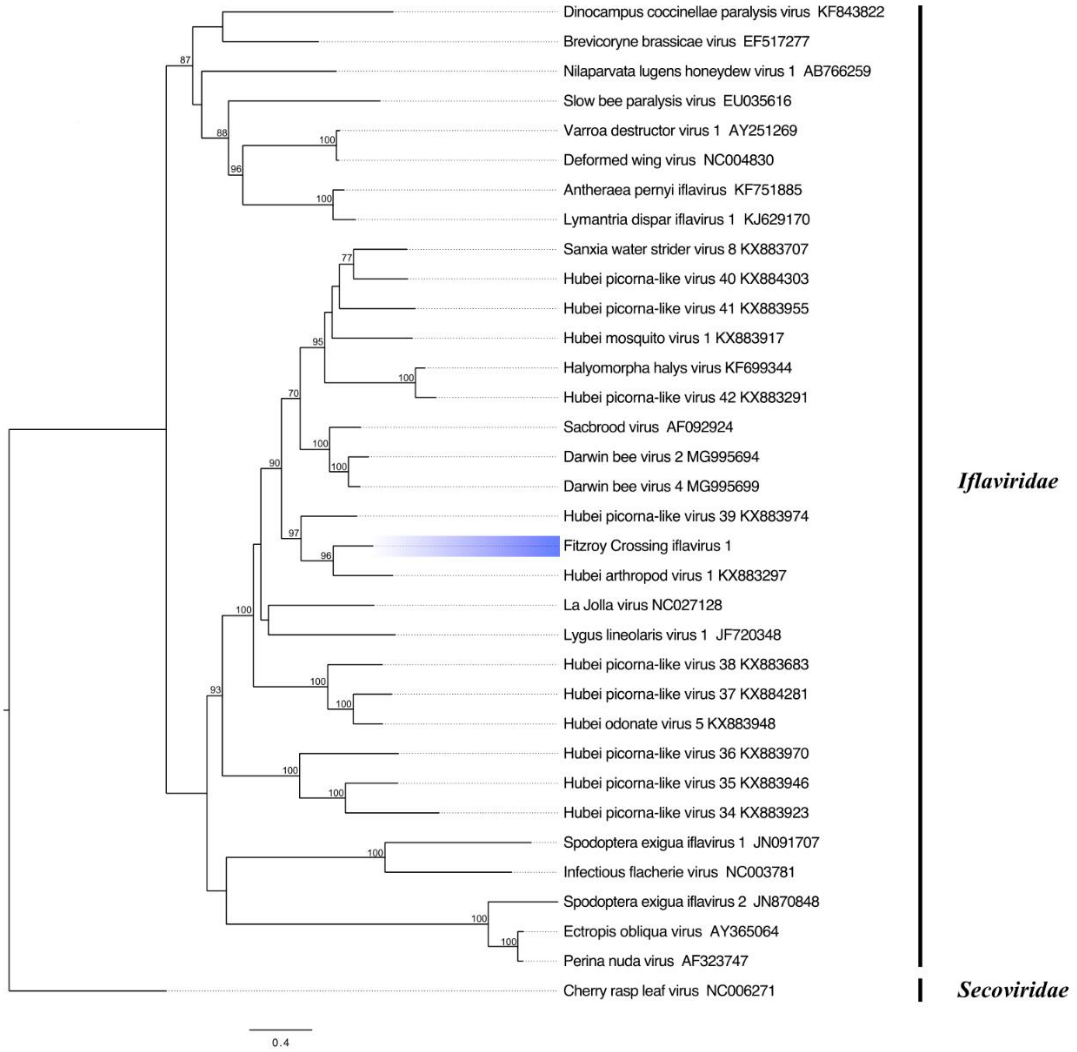

Figure S1. Phylogenetic analysis of Fitzroy Crossing iflavirus 1. The maximum likelihood tree was constructed usind RdRp protein sequence. The scale bar represents units of substitutions per site and bootstrap support va;ues are displayed when greater that 70%. Viruses identified in this study are highlighted in blue.

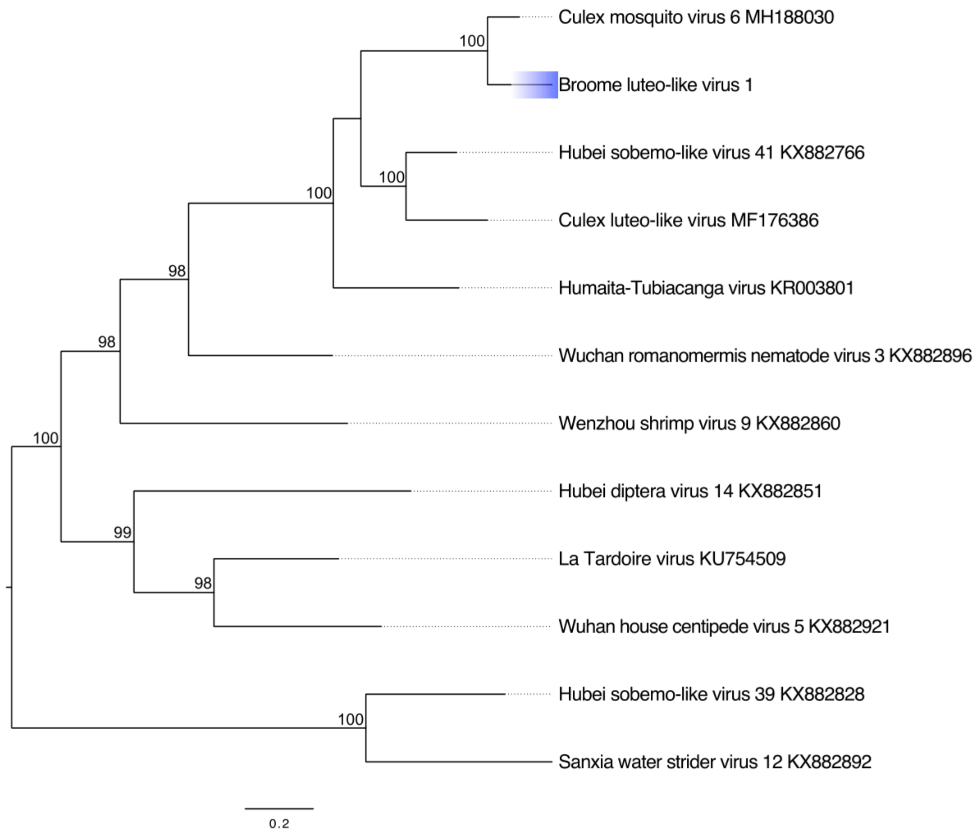

Figure S2. Phylogenetic analysis of Broome luteo-like virus. The maximum likelihood tree was constructed usind RdRp protein sequence. The scale bar represents units of substitutions per site and bootstrap support values are displayed when greater that 70%. Viruses identified in this study are highlighted in blue.

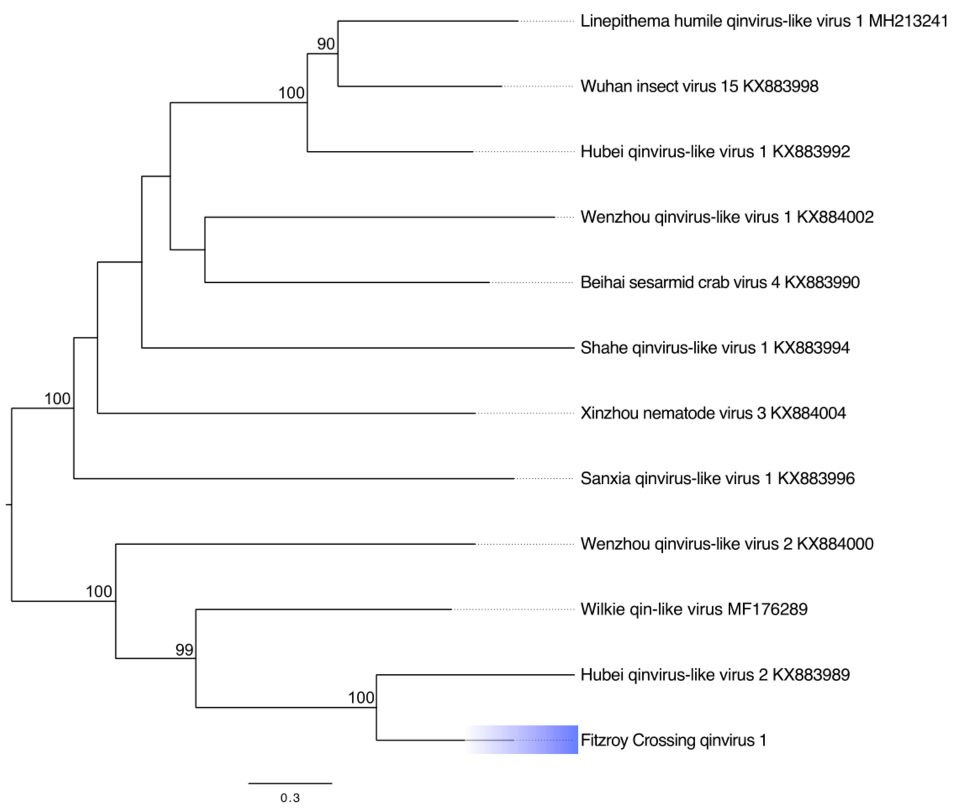

Figure S3. Phylogenetic analysis of Fitzroy Crossing qinvirus 1. The maximum likelihood tree was constructed using RdRp protein sequence. The scale bar represents units of substitutions per site and bootstrap support values are displayed when greater that 70%. Viruses identified in this study are highlighted in blue.

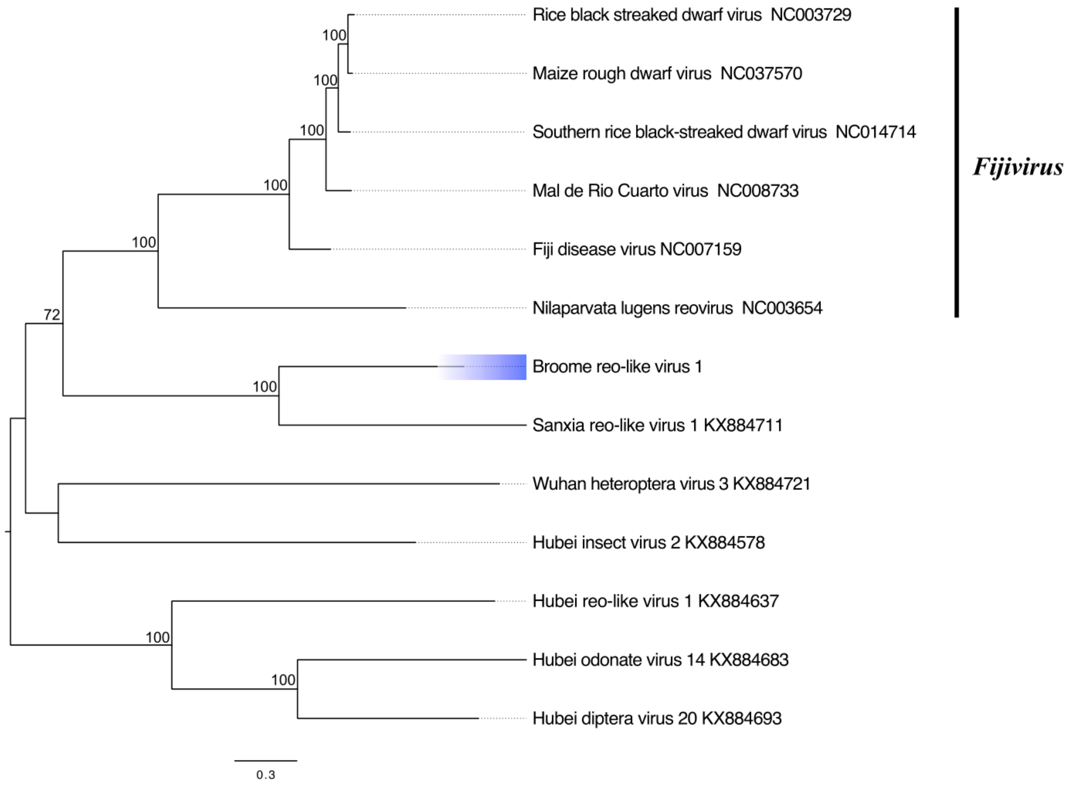

Figure S4. Phylogenetic analysis of Broome reo-like virus 1. The maximum likelihood tree was constructed using RdRp protein sequence. The scale bar represents units of substitutions per site and bootstrap support values are displayed when greater that 70%. Viruses identified in this study are highlighted in blue.

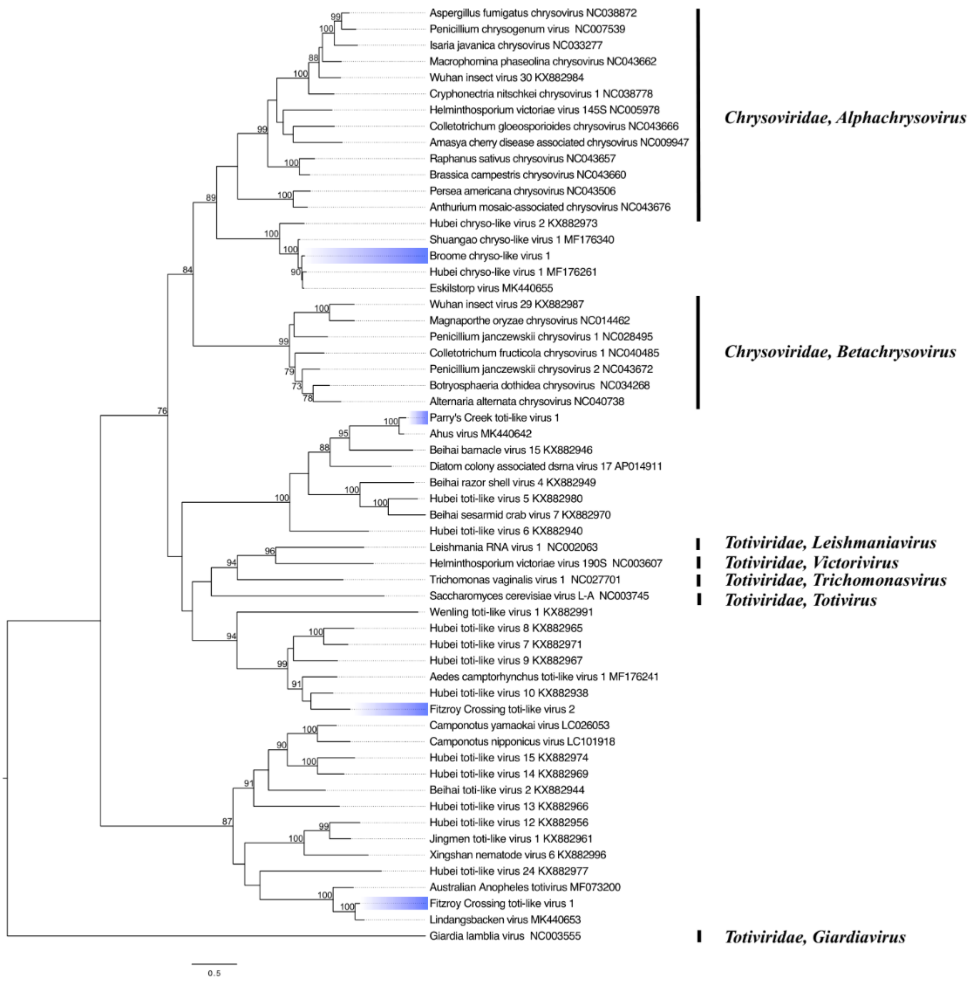

Figure S5. Phylogenetic analysis of chryso-like and toti-like viruses. The maximum likelihood tree was constructed using RdRp protein sequence. The scale bar represents units of substitutions per site and bootstrap support values are displayed when greater that 70%. Viruses identified in this study are highlighted in blue.

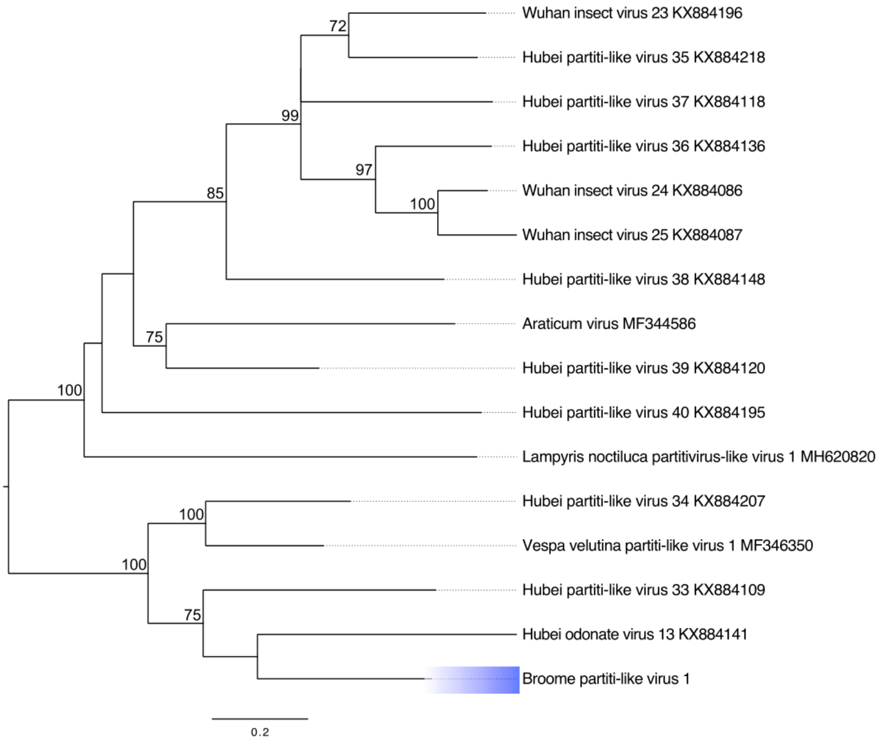

Figure S6. Phylogenetic analysis of Broome partiti-like virus 1. The maximum likelihood tree was constructed using RdRp protein sequence. The scale bar represents units of substitutions per site and bootstrap support values are displayed when greater that 70%. Viruses identified in this study are highlighted in blue.
